# Undifferentiated carcinoma of the transverse colon with rhabdoid features that developed during treatment of non-small cell lung carcinoma with pembrolizumab: a case report

**DOI:** 10.1186/s40792-020-00963-1

**Published:** 2020-08-03

**Authors:** Yuya Ashitomi, Mitsuhiro Yano, Michihisa Kono, Takefumi Suzuki, Ichiro Kawamura, Shinji Okazaki, Yukinori Kamio, Osamu Hachiya, Yuka Urano, Fuyuhiko Motoi

**Affiliations:** 1 First Department of Surgery, Yamagata University Faculty of Medicine, 2-2-2, Iida-Nishi, Yamagata City, Yamagata 990-9585 Japan; 2 Department of Pathological Diagnostics, Yamagata University Faculty of Medicine, 2-2-2, Iida-Nishi, Yamagata City, Yamagata 990-9585 Japan

**Keywords:** Colorectal cancer, Undifferentiated carcinoma, Rhabdoid tumor, Pembrolizumab

## Abstract

**Background:**

Undifferentiated carcinoma of the colon is rare, and its prognosis is very poor. We report a case of undifferentiated carcinoma of the colon with rhabdoid features developed during treatment of non-small lung carcinoma (NSCLC) with pembrolizumab.

**Case presentation:**

A 58-year-old man was diagnosed with transverse colon cancer during chemotherapy with pembrolizumab for NSCLC. Laparoscopic right hemicolectomy was performed. The histopathological diagnosis was undifferentiated carcinoma with rhabdoid features and lymph node metastasis. Immunohistochemically, programmed death ligand 1 (PD-L1) showed positivity. The microsatellite instability (MSI) status was low. He continued to receive pembrolizumab for NSCLC, and there have been no signs of colon cancer recurrence and progression of NSCLC for 15 months.

**Conclusion:**

We present the case of an undifferentiated carcinoma of the transverse colon with rhabdoid features. The development of the tumor with the expression of PD-L1 during pembrolizumab might have been associated with the low MSI.

## Background

Undifferentiated carcinoma with rhabdoid features is rare and confers poor prognosis [1]. These tumors have been reported to occur at several sites, such as the central nervous system, gastrointestinal tract, heart, breast, and urinary tract [2, 3]. Pembrolizumab is an immune checkpoint inhibitor (ICI) that targets programmed death-1 (PD-1) [4]. It is used for the treatment of many types of cancers, including non-small cell lung cancer (NSCLC). We report a case of undifferentiated carcinoma of the transverse colon with rhabdoid features developed during treatment of NSCLC with pembrolizumab.

## Case presentation

**Fig. 1:**
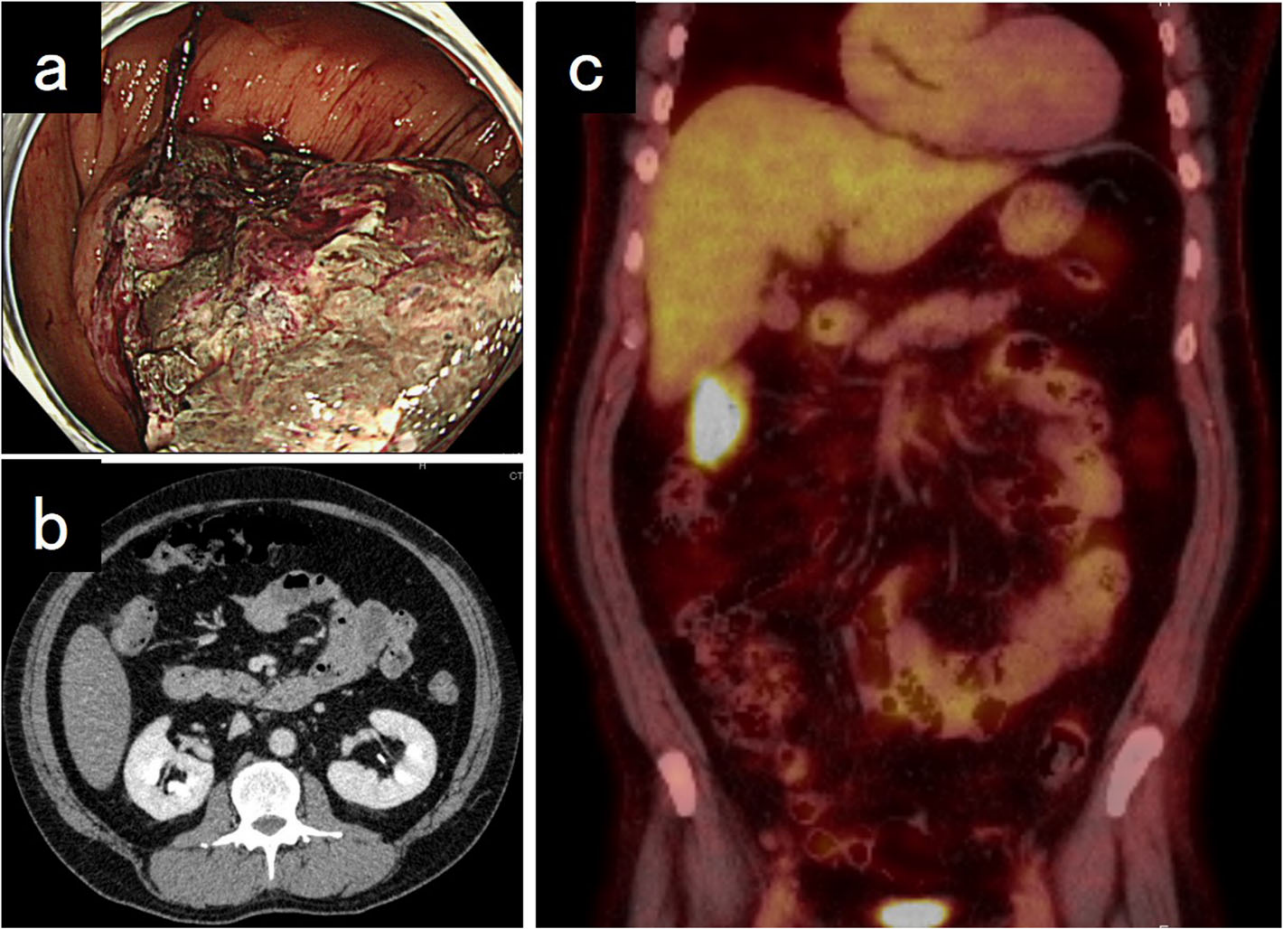
Image of the transverse colon cancer. Colonoscopy showed a type 3 tumor with necrotic tissue (**a**). Contrast-enhanced CT did not indicate colon cancer (**b**). Positron emission tomography showed an accumulation of ^18^F-fluorodeoxyglucose in the colon (**c**)

**Fig. 2:**
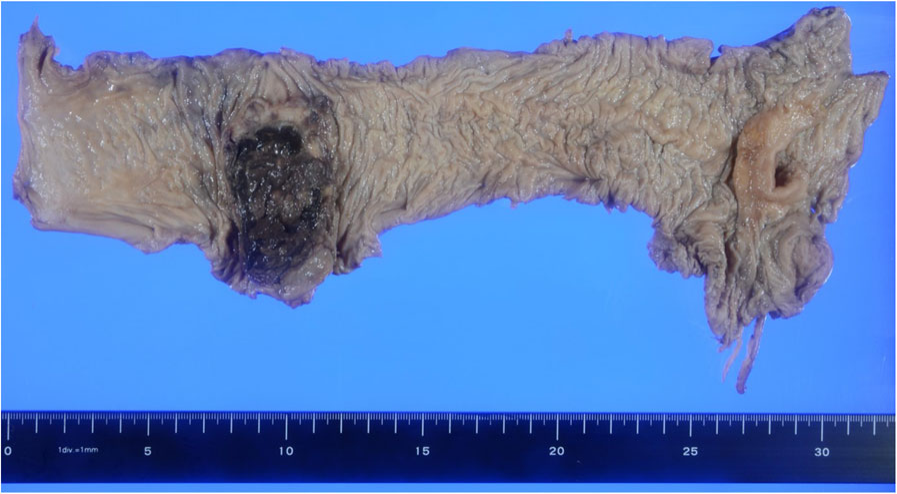
Macroscopic findings of the resected specimen. The tumor size was 75 × 46 mm

**Fig. 3:**
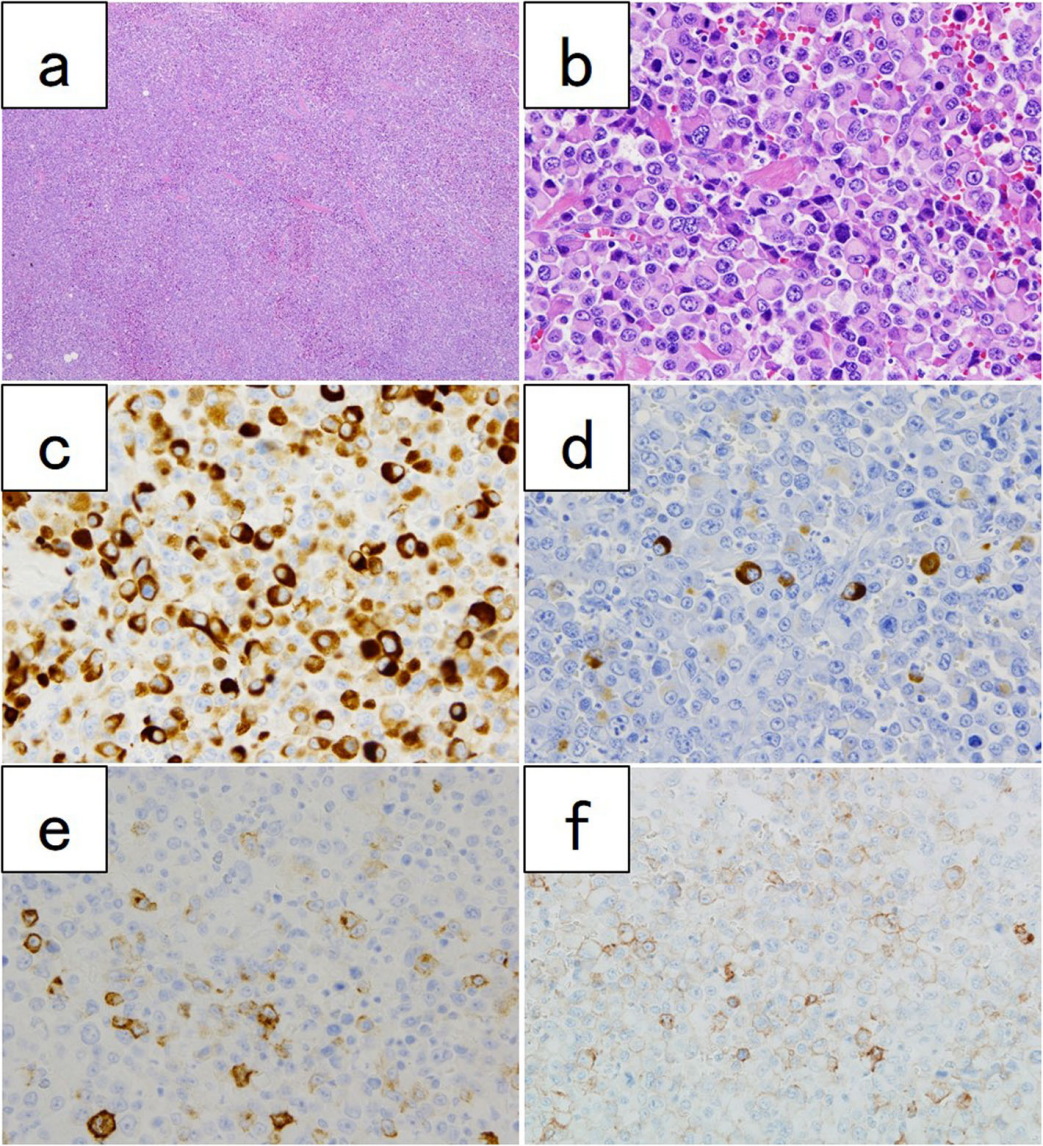
Histological and immunohistochemical studies. Hematoxylin and eosin staining of tumor cells with rhabdoid cells (**a**, × 40, **b** × 400). AE1/AE3 positivity (**c**). CAM 5.2 focally positivity (**d**). EMA focally positivity (**e**). Programmed death-ligand 1 positivity (**f**)

A 58-year-old man was receiving pembrolizumab as a 1st line treatment for NSCLC (showing differentiation into adenocarcinoma and squamous cell carcinoma) and multiple bone metastases for 9 months. The patient presented with anemia and bloody stools. Colonoscopy revealed a type 3 lesion at the transverse colon, and the biopsy showed an undifferentiated carcinoma. Computed tomography showed multiple swollen lymph nodes along the superior mesenteric artery. ^18^F-fluorodeoxyglucose positron emission tomography was performed for disease evaluation, and accumulation was observed in the right colon (maximum standardized uptake value of 22) (Fig. 1). The preoperative diagnosis was cT3N1bM0 stage IIIB (union for international cancer control (UICC) 8th edition) locally advanced transverse colon cancer. We performed laparoscopic right hemicolectomy with lymphadenectomy. He was discharged on the 10 days after the surgery without postoperative complications. The resected specimen showed a tumor measuring 75 × 46 mm (Fig. 2). Histologically, undifferentiated cancer cells and diffuse invasion of rhabdoid tumors were observed. Immunohistochemically, the tumor cells tested positive for AE1/AE3 and focally positive for CAM5.2 and epithelial membrane antigen. Programmed death-ligand 1 (PD-L1) tested positive (Fig. 3). Synaptophysin, chromogranin A, thyroid transcription factor-1, surfactant protein A, cytokeratin 5/6, p40, S-100P, D2-40, leukocyte common antigen, α-smooth muscle actin, desmin, calponin, h-caldesmon, cytokeratin 20, and E-cadherin were all tested negative. The microsatellite instability (MSI) status was low. The final diagnosis was undifferentiated carcinoma with rhabdoid features and lymph node metastasis (pT3N2aM0 Stage IIIB, UICC 8th edition). He continued to be received pembrolizumab for NSCLC. There have been no signs of colon cancer recurrence and progression of NSCLC for 15 months.

## Discussion

Pembrolizumab is an ICI, a relatively new drug. Pembrolizumab is a humanized monoclonal Immunoglobulin G4 kappa antibody that prevents PD-1 from binding with two ligands: PD-L1 and programmed death ligand 2 [4]. Pembrolizumab has shown significant efficacy for PD-L1-positive NSCLC in clinical trials [5–7], and the Food and Drug Administration in USA approved pembrolizumab for the treatment of metastatic NSCLC. Pembrolizumab is now used for not only NSCLC but also for various tumors such as melanoma and MSI-high solid tumors [4, 8–12]. In present case, NSCLC with bone metastasis exhibited PD-L1 positivity. The patient received pembrolizumab, and the NSCLC was controlled without disease progression. Unlike other drugs, most adverse events (AEs) of ICIs are thought to be immune-related AEs. ICIs affect immune system, resulting in immune-related AEs such as hypothyroidism, diabetes mellitus, and pneumonitis [4].

Rhabdoid tumors were first reported in young children with renal tumors [13, 14]. This tumor was later reported at several sites, such as central nervous system, gastrointestinal tract, heart, breast, and urinary tract [15]. Rhabdoid tumors are histologically characterized by the unique morphological features of proliferating rhabdoid cells, which have abnormally located large nuclei, prominent nucleoli, and typical eosinophilic inclusion of aggregated intermediate filament [3, 13]. Rhabdoid tumors are classified into two types: composite and pure. Both show similar morphologic features and immunohistochemical results [16]. There is no standard therapeutic protocol for these tumors, and previous reports indicate a poor prognosis [17, 18]. Moussaly et al. reported that most patients with colorectal rhabdoid tumors died within 6 months after surgery [19]. However, there were few reports of cases involving long-term survival of more than 1 year [1, 16, 20, 21]. The histogenesis of rhabdoid tumor is uncertain. In present case, the colonic tumor that developed during treatment with pembrolizumab was undifferentiated carcinoma despite PD-L1 positivity. In NSCLC, PD-L1 expression is associated with the response of pembrolizumab [5]. Czink E, et al. reported the MSI-high biliary tract cancer with a lack of expression of PD-L1 may be successfully treated by ICIs [22]. Some reports suggest that there is no association between the treatment effects of anti-PD-1/PD-L1 agents and the value of PD-L1 [23]. The therapeutic effect of pembrolizumab for the colon cancer was insufficient because the tumor had been progressed during the treatment in presented case. However, it could be speculated that pembrolizumab has prevented distant recurrence of this highly malignant tumor for more than 1 year. Further investigations would provide insight into the clinicopathological feature of this highly malignant tumor and its association with the actions of ICIs. To the best of our knowledge, there have been no reports of carcinoma that have developed during treatment with ICIs.

## Conclusion

We present a 58-year-old man who was found to have an undifferentiated carcinoma of the transverse colon with rhabdoid features. The development of this PD-L1 expressed tumor during pembrolizumab treatment might have been associated with the low tumor mutational burden indicated by low MSI.

## Data Availability

Not applicable.

## Abbreviations

ICI: Immune checkpoint inhibitors
PD-1: Programmed death-1
NSCLC: Non-small cell lung cancer
UICC: Union for international cancer control
PD-L1: Programmed death-ligand 1
MSI: Microsatellite instability
AEs: Adverse events
